# Supplementary material for: A Comprehensive Assessment of Ultraviolet-Radiation-Induced Mutations in Flammulina filiformis Using Whole-Genome Resequencing
Source: J Fungi (Basel). 2024 Mar 20;10(3):228. doi: 10.3390/jof10030228 (PMC10971301; doi:10.3390/jof10030228)
Supplement: Supplementary file 1 [file jof-10-00228-s001.zip › Supplementary Material S8/KEGG annotation/out/64381550635650.os/KO/out_map/map04662.html]

KEGG PATHWAY: B cell receptor signaling pathway - Reference pathway


|  |  |
| --- | --- |
| **B cell receptor signaling pathway - Reference pathway** |  |

[
Pathway menu
| Organism menu
| Pathway entry
| Show description
| User data mapping
]

B cells are an important component of adaptive immunity. They produce and secrete millions of different antibody molecules, each of which recognizes a different (foreign) antigen. The B cell receptor (BCR) is an integral membrane protein complex that is composed of two immunoglobulin (Ig) heavy chains, two Ig light chains and two heterodimers of Ig-alpha and Ig-beta. After BCR ligation by antigen, three main protein tyrosine kinases (PTKs) -the SRC-family kinase LYN, SYK and the TEC-family kinase BTK- are activated. Phosphatidylinositol 3-kinase (PI3K) and phospholipase C-gamma 2 (PLC-gamma 2) are important downstream effectors of BCR signalling. This signalling ultimately results in the expression of immediate early genes that further activate the expression of other genes involved in B cell proliferation, differentiation and Ig production as well as other processes.

|  |  |
| --- | --- |
| Reference pathway | 100% |
